# Targeted Therapy for Orofacial Pain: A Novel Perspective for Precision Medicine

**DOI:** 10.3390/jpm13030565

**Published:** 2023-03-22

**Authors:** Swarnalakshmi Raman, Daisuke Ikutame, Kazuo Okura, Yoshizo Matsuka

**Affiliations:** Department of Stomatognathic Function and Occlusal Reconstruction, Graduate School of Biomedical Sciences, Tokushima University, Tokushima 770-8504, Japan

**Keywords:** orofacial pain, pain, precision medicine, personalized medicine, personalized care, neuropathic pain, targeted therapy, literature review

## Abstract

Orofacial pain (OFP) is a dental specialty that includes the diagnosis, management and treatment of disorders of the jaw, mouth, face, head and neck. Evidence-based understanding is critical in effectively treating OFPs as the pathophysiology of these conditions is multifactorial. Since OFP impacts the quality of life of the affected individuals, treating patients successfully is of the utmost significance. Despite the therapeutic choices available, treating OFP is still quite challenging, owing to inter-patient variations. The emerging trends in precision medicine could probably lead us to a paradigm shift in effectively managing the untreatable long-standing pain conditions. Precision medicine is designed based on the patient’s genetic profile to meet their needs. Several significant relationships have been discovered based on the genetics and genomics of pain in the past, and some of the notable targets are discussed in this review. The scope of this review is to discuss preclinical and clinical trials that include approaches used in targeted therapy for orofacial pain. Future developments in pain medicine should benefit from current trends in research into novel therapeutic approaches.

## 1. Introduction

Orofacial pain (OFP) is the term used to define pain affecting the hard and soft tissues of the face and/or oral cavity [1,2]. Pain is a stimulus modality, it is subjective and serves as a warning sign for response to damaged tissue in the body [3,4]. The prevalence of OFP is reported to be between 16.1 and 33.2%, out of which 10% is considered to contribute to chronic orofacial pain [5]. OFP can range from a straightforward intraoral pain affecting the teeth or periodontium to more extensive involvement of cranial nerves or lesions or sometimes OFP also resembles headaches [6]. The pathophysiology of these OFPs is multifactorial and is being extensively studied to understand the underlying mechanism. OFP lasting more than three months is defined as chronic OFP. Chronic pain is a disease state, that outlasts even the time of healing when associated with illness or injury. Chronic pain widely relies on a multidisciplinary approach [7]. Of paramount importance is the effective treatment of patients as OFP affects the quality of life of the affected individuals. Despite the availability of a multitude of therapeutic options, effective treatment of OFP remains quite challenging in a wide variety of cases. This could be owed to the inter-patient variations.

The emerging trends in “precision medicine” might lead us to a paradigm shift in effectively managing untreatable long-standing pain conditions. Precision medicine refers to the tailor-made optimization of therapeutics to individuals, or a particular group of patients based on their genetic or molecular profiling. In other words, it is the customization of medical management rather than “one drug fitting all”. It is occasionally also called personalized medicine or personalized care. Currently, precision medicine is rapidly evolving in the field of oncology and rare genetic diseases [8,9,10].

The heterogeneity of orofacial pain and the inter-patient variations in analgesic treatment are fundamental to focusing on individualized medicine. An example for such a scenario is that some patients may not respond to morphine therapy, however they may respond when changing to other opioids. There seems to be a linkage between the genetic makeup and analgesic response [11,12,13].

## 2. History of Precision Medicine

The idea of patient-centered care has existed for ages, dating back to Hippocrates (460–370 BCE), who accurately stated “It’s far more important to know what person the disease has than what disease the person has” [14]. The term personalized medicine was indeed already reported in an article by W.M. Gibson in 1971, who envisioned the family physician’s role as a scientist–physician. He precisely stated that the family physician “Within a few years will likely have available to him a computer programmed for medicine providing him with a great store of knowledge literally at his fingertips” [15].

Over the years, several terms have been used to describe individualized therapy, and in the past decade the preferred term has been “precision medicine”, shifted from personalized medicine. The National Research Council chose the word precision medicine due to concerns that that personalized medicine may be interpreted incorrectly as unique or individualized treatment for each person. Indeed, the term “precision medicine” has been used by Wasi in 1997 in the discussion of the future of genomic medicine [16]. He wrote that human genomics is fundamental for diagnosis, treatment, prognosis and prevention, it will give rise to “predictive-preventive medicine and precision medicine”. However, it was only in 2015, after Barack Obama mentioned about a “precision medicine initiative”, that it became a preferred term [17].

## 3. The Need for Advancement of Precision Medicine in Pain Management

Chronic pain is a global burden, the current treatments available are only selectively effective or have side effects. Orofacial pain is one of the severe pains affecting approximately 25% of the population [5,18]. The type of orofacial pain is classified based on the pathophysiology and symptoms. The classification has been published by the International Classification of Orofacial Pain (Table 1) [6]. Non-odontogenic variants of orofacial pain are difficult to diagnose and usually misdiagnosed. Hence, for appropriate diagnosis and effective management, the underlying mechanism has been researched widely in recent decades. Despite abundant treatment options prevailing, the management of orofacial pain still remains unmet in some cases.

Over the years, there has been tremendous progress in the field of genetics and understanding the genomics of pain. Significant links of pain have been discovered and researched in the past, however these are still under a “trial and error” state, and need further validation through animal and human studies. The current treatment is based on pharmacotherapy, and advancements in genetics have paved way for precision medicine. Precision medicine aims to make better diagnoses and improve patient management by understanding more about the human condition. The potential for developing better medications based on a greater understanding of the pathophysiology of illnesses is a topic of considerable discussion. In precision medicine, pain management uses techniques to evaluate every patient individually, determine their risk profile for experiencing exaggerated pain or the emergence of chronic pain, and then refine therapeutic approaches to target particular pathological processes underlying chronic pain. Current trends in exploring the new therapeutic avenues in pain management should aid in improving the precision medicine for pain in the future.

## 4. Precision Medicine Targeting Orofacial Pain

Conventional treatment involves the selection of drugs based on the cause of pain, the pain characteristics, patient history, age, gender, etc. Further, the dose is modified based on the patient response of pain relief and side effects. The existing lacunae in this kind of treatment is the failure to consider genomic criteria in the treatment strategy. Hence, there is limited efficacy in these routine pharmacotherapies, further leading to the need for precision medicine.

**Figure 1:**
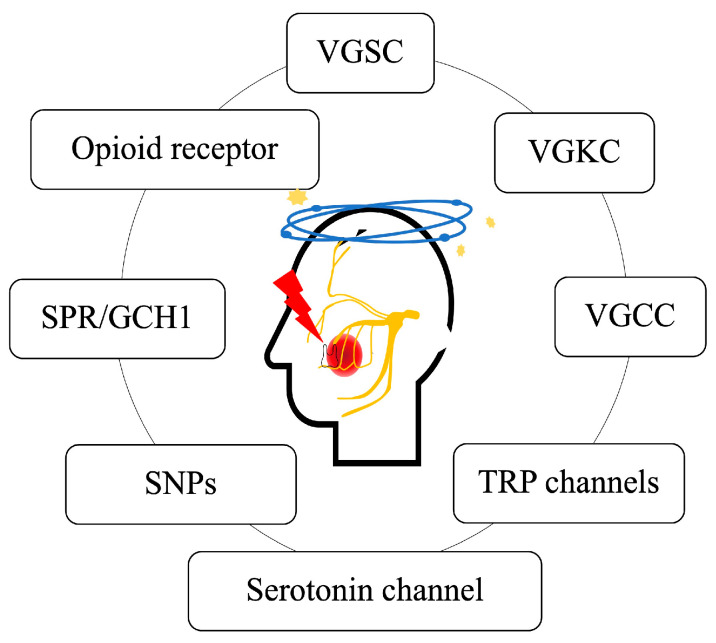
Potential targets of orofacial pain. VGSC: voltage-gated sodium channel; VGKC: voltage-gated potassium channel; VGCC: voltage-gated calcium channel; TRP: transient receptor potential channels; SNP: single-nucleotide polymorphism; SPR: sepiapterin reductase; GCH1: GTP cyclohydroxylase 1.

Precision medicine is thoroughly based on the genetic makeup of the patient, specially tailored based on the patient needs. The development of precision medicine for pain is relatively slow when compared to that of other fields. Genetic analysis together with pharmacological analysis can predict the efficacy of drugs in a patient-specific manner. Based on the genetics and genomics of orofacial pain, several important links have been identified in the past and some of the notable targets are shown in Figure 1.

Ion channels are classified based on what closes and opens the channel and the nature of ions passing through the pores (gates). We have discussed below the role of the various gated channels.

### 4.1. Voltage-Gated Sodium Channel

Voltage-gated sodium channels (VGSCs) function as hetero-multimeric proteins that consist of an α subunit and an auxiliary β subunit. There are nine distinct VGSCs subtypes: Nav1.1 to Nav1.9. The Nav1.3, Nav1.7, Nav1.8 and Nav1.9 channels have been discovered to have contributed to neuropathic pain [19]. In the ongoing research trend, sodium channels are the key targets for treating neuropathic pain. However, currently available drugs are of limited efficacy and poor tolerability. For further promising results, screening strategies to identify molecules binding to distinct subtypes would resolve the concerns.

### 4.2. Voltage-Gated Potassium Channel

The family of voltage-gated potassium channels (VGKC), often known as K_v_ channels, has 12 different members. K_v_ channels play a crucial role in regulating neuronal excitability by participating in action potential repolarization and damping membrane depolarization. Compared to sodium channel blockers, drugs that target potassium channels have received significantly less research [20]. A more detailed understanding is necessary on medications that alter potassium channels since they may be a potent addition to the current options for alleviating neuropathic pain. Identification of specific sensory defects and genetic profiling of patients may predict the therapeutic benefit of K_v_ channels [20,21].

### 4.3. Voltage-Gated Calcium Channel

The voltage-gated calcium channels (VGCC) are a group of ion channels found in the membrane of excitable cells, such as muscle, neurons, glial cells, etc. They are broadly classified into high-voltage-activated and low-voltage-activated channels. There are several different subunits (α1, α2δ, β1–4, and γ) [22,23]. VGCCs are well-known pain signal mediators in primary afferent neurons [24]. Even though calcium channels have been studied as a possible therapeutic target for more than 20 years, these agents are not reported yet to be effective in clinical management of pain [25].

### 4.4. Transient Receptor Potential Channels

Transient Receptor Potential (TRP) channels are a type of cationic channels that function as signal transducers by modifying intracellular calcium or membrane potential. The TRP channel superfamily is classified into six subfamilies: TRPC (Canonical), TRPV (Vanilloid), TRPM (Melastatin), TRPA (Ankyrin), TRPML (Mucolipin) and TRPP (Polycystic) [26]. These channels have been identified to have a role in a range of pain modalities, including inflammatory pain, neuropathic pain, visceral pain and pain related to specific pathological disorders, such as cancer or migraine [27]. TRPV1 and TRPA1 have been explored due to their antinociceptive properties and were found to be beneficial in relieving cancer-derived pain [28]. TRP channels are improving their importance in different areas, becoming suitable as promising candidates for precision medicine.

### 4.5. Serotonin-Gated Ion Channels

Serotonin-gated ion channels are opened directly by the neurotransmitter acetylcholine, glycine or glutamate. Serotonin directly activates 5-hydroxytryptamine (5-HT3) channels, which are nonselective cationic ion channels [29]. The role of 5-HT in neuropathic pain has been reported in several studies but the specific receptors have not been studied [30].

### 4.6. Single Nucleotide Polymorphisms

Single nucleotide polymorphisms (SNP) are the most common type of genetic variation among people. They aid in predicting a person’s susceptibility to specific medications, tolerance to environmental factors such as toxins, and risk of contracting diseases. SNPs can also be used to monitor how disease-related genetic variations are passed down across families. Techniques based on gene therapy and the regulation of epigenetic alterations broaden future options to enhance the understanding of pain mechanisms and their treatment by novel medicines, opening doors to precision medicine [31]. Some recent promising SNPs ideal for targeted therapy have been discussed below.

- Solute carrier family 17 member 9 (SLC17A9) and purinergic receptor P2Y12 (P2RY12) have been reported to be associated with neuropathic pain. Further studies are needed for understanding the detailed mechanisms of pain signal transduction in humans [32].
- Catechol-O-methyltransferase [COMT] is a metabolic enzyme found primarily in postsynaptic neurons and glial cells. Previous reports have shown the participation of COMT in the regulation of neurotransmitters such as dopamine, noradrenaline and adrenaline related to pain [33,34,35,36]. The literature shows evidence of the association of COMT with pain modulation in temporomandibular disorders, which would aid in progressing toward precision medicine.

### 4.7. Sepiapterin Reductase/GTP Cyclohydrolase 1

Sepiapterin reductase (SPR) is a prime enzyme in the synthesis of tetrahydrobiopterin (BH4). GTP cyclohydroxylase 1 catalyzes the initial and rate-limiting steps in the synthetic pathway of BH4. BH4 plays a major role in cardiovascular function, mood, inflammation and neurotransmission. An association between BH4 increase and axonal injury has been reported previously [37]. Further, the association between GCH1 and orofacial pain is yet to be explored. SPR is emerging as a novel therapeutic target for treating neuropathic pain [38].

### 4.8. Opioid Receptors

Opioid receptors are a group of inhibitory G-protein-coupled receptors with opioids as ligands. There are three types of opioid receptors, designated as mu, delta and kappa. Opioid analgesics are well established for their use in analgesia, including their abusive risks [39]. Hopefully, the recent knowledge of opioid analgesic drugs may aid in new drug development for newer therapeutic approaches in precision medicine [40].

The preclinical and clinical trials reported in recent years on each potential target, specifically on the orofacial region, have been summarized in Table 2 and Table 3.

## 5. Challenges

Translational issues in precision medicine, particularly in neuropathic pain, are one of the main challenges. New drugs fail to be implemented in clinical practice due to the translational gap from traditional animal models to clinical application. Although there has been a persistent desire to focus on precision medicine as a method of treating the condition’s underlying cause, it is exceptionally rare for a disorder to originate from just a single gene mutation [63]. In pain, more frequently, mutations are characterized in sodium channels. Despite several novel blockers targeting these sodium channels, the translation of these laboratory results to practical day-to-day use in humans is not generally available [64]. The major reason for the translational gap is failure in clinical studies due to inappropriate endpoint selection for validation [63]. For example, 5HT3-antagonist when used for neuropathy patients showed positive results when ongoing pain [65] was evaluated, whereas it showed negative results when dynamic pain was evaluated [66]. Hence, it is necessary to pick an ideal endpoint for validation of the efficacy in patients.

## 6. Multifaced Perspectives

**Figure 2:**
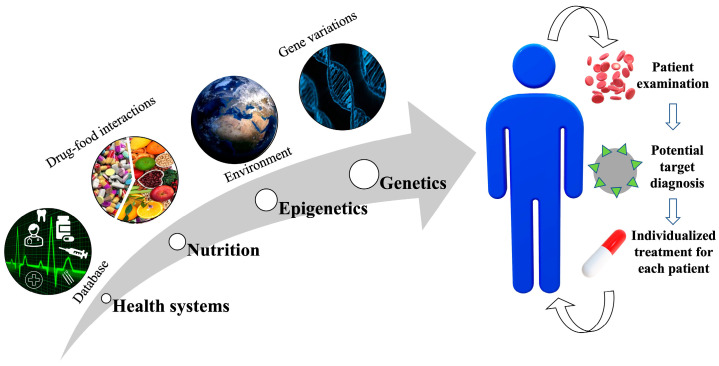
Scheme for various perspectives for effective precision medicine. Various factors include databases by health systems, nutrition, epigenetics and genetics. A multifaced approach would aid in the diagnosis of the potential target, leading to an effective individualized treatment for each patient.

There are various aspects to be considered for effective precision medicine. Figure 2 shows a schematic representation of the multifaced perspectives.

### 6.1. Health Systems

The database systems are essential for clinical evaluation, decisions, monitoring and to improve efficiency. Large-scale data access is essential for determining the suitable precision medicine for an individual [67].

### 6.2. Nutrition

The drug–food interactions play a vital factor as they may often result in malabsorption of the medicine. Hence, the diet of the individual is to be considered in precision medicine [68].

### 6.3. Epigenetics

Depression, stress, nerve damage and alcohol intake all cause epigenetic alterations that both directly and indirectly affect pain and the effects of drugs [69,70,71,72].

### 6.4. Genetics

The application of genetics in clinical decision making has existed for many years. For instance, keeping track of a patient’s family history provides insights into heritable patterns that indicate disease susceptibility or perhaps help choose the appropriate treatment regimen. We must investigate how common and rare genetic variations prevalent across many regions of the world affect health and treatment response if personalized medicine is to be credible and applicable to the global communities [73].

## 7. Future Directions

Precision medicine is of great ongoing interest. Immense research has been unraveling to tailor therapies to benefit the individual patient with maximum efficacy and reduced adverse effects. However, we must understand how common and rare genetic variations present in various geographic locations affect health and drug response if personalized medicine is to be meaningful and applicable to the population of the entire planet. For the implementation of precision medicine in clinical practice, there is a need for a better understanding of these potential targets’ (ion channels and TRP channels) role in pain pathogenesis [45,48]. The association of pain-related single-nucleotide polymorphism with the clinical phenotypes is essential for targeted therapy implementation [32,50]. The importance of GTP cyclohydrolase 1, sepiapterin reductase and catechol-O-methyltransferase has been extensively established in the context of pain mechanisms, although clinical trials in orofacial research are still required [37,38,74]. Despite an array of pharmacological drugs, sodium channel modulators are not yet pharmacotherapeutic for neuropathic pain; future research should consider the heterogeneity in pain phenotypes [55,56,75]. Furthermore, genetic testing needs to be reasonably priced for precision medicine to be effectively implemented. Large-scale studies on inherited pain would provide better perspectives in a broader population. Treatment strategies to target specific channel by selective blocking would aid in enhanced therapeutics in pain with reduced side effects. The exploration of drugs with new mechanisms based on genomic analysis would aid in more effective treatment. Enhanced research in pain genetics can widen the scope for individualized treatment for some rather than all.

## 8. Conclusions

Precision medicine tendencies might create a turning point in treating chronic pain illnesses, currently incurable in some patients. Precision medicine for orofacial pain is still in the preliminary stage despite several novel and potential targets being reported in the past. Overcoming the translational gaps and additionally considering standard protocols for screening would be promising for the success in precision medicine of the orofacial region in the near future.

## Figures and Tables

**Table 1:** Classification describing the diagnostic criteria.

1.	Orofacial pain attributed to disorders of dentoalveolar and anatomically related structures 1.1 Dental pain 1.2 Oral mucosal, salivary gland and jawbone pains
2.	Myofascial orofacial pain 2.1 Primary myofascial orofacial pain 2.2 Secondary myofascial orofacial pain
3.	Temporomandibular joint (TMJ) pain 3.1 Primary temporomandibular joint pain 3.2 Secondary temporomandibular joint pain
4.	Orofacial pain attributed to lesion or disease of the cranial nerves 4.1 Pain attributed to lesion or disease of the trigeminal nerve 4.2 Pain attributed to lesion or disease of the glossopharyngeal nerve
5.	Orofacial pains resembling presentations of primary headaches 5.1 Orofacial migraine 5.2 Tension-type orofacial migraine 5.3 Trigeminal autonomic orofacial pain 5.4 Neurovascular orofacial pain
6.	Idiopathic orofacial pain 6.1 Burning mouth syndrome 6.2 Persistent idiopathic facial pain 6.3 Persistent idiopathic dentoalveolar pain 6.4 Constant unilateral facial pain with additional attack
7.	Psychosocial assessment of patients with orofacial pain

**Table 2:** Potential targets for orofacial pain—preclinical trials.

Potential Targets	First Author, Year, Country	Animal and Experiment Condition	Results/Outcome	Opinion
VGSC	Eriksson et al., 2005, Sweden [41]	Rat—Partial ischemic injury to the infraorbital branch of the trigeminal nerve.	The beta 3 subunit was significantly upregulated, whereas the mRNAs for Na_v_1.8 and Na_v_ 1.9 were reduced in terms of number of neurons and intensities.	Designing a sodium channel subtype-specific blocker from these observations would be helpful for orofacial neuropathic pain therapeutics.
	Luiz et al., 2015, UK [42]	Na_v_1.9^–/–^ and Na_v_1.9^+/+^ mice—Unilateral constriction of the infraorbital nerve.	Reported the role of Na_v_1.9 sodium channel in the development of thermal and mechanical hypersensitivity.	Regulation of Na_v_1.9 channel should be investigated for the treatment of orofacial neuropathic pain.
VGKC	Madrid at al., 2009, Spain [43]	Neonatal (P1–P6) and adult OF1 mice.	Excitation and inhibition of K_v_1 channels modulate the thermosensitive channels.	Suggests the possibility of K_v_1 channels in alleviating peripheral gating of cold-evoked discomfort and pain.
	Kanda et al., 2021, AL, USA [44]	Rat—Infraorbital nerve constriction injury (chronic model).	Dysfunction of K_v_4.3 channels in sensory neurons of trigeminal ganglion exhibit cold hypersensitivity in orofacial regions leading to neuropathic pain.	Selective K_v_4.3 activators may be clinically useful to alleviate trigeminal neuropathic pain.
VGCC	Montera et al., 2021, NM, USA [45]	Mice—Foramen rotundum inflammatory Constriction of trigeminal infraorbital nerve.	Upregulation of Ca_v_3.3 in injury model and Ca_v_3.3 blocking significantly reduced allodynia and attenuated neuropathic pain.	Blocking Ca_v_3.3 function may be effective in the treatment of trigeminal neuropathic pain. The blocking of Ca_v_3.3 may be more effective in females.
	Gambeta et al., 2022, Canada [46]	Mice—Constriction of infraorbital nerve.	Antihyperalgesia was the outcome. With the use of a selective T-type calcium channel blocker (Z944). It had no impact in Ca_V_3.2^−/−^ mice.	T-type calcium channel (Ca_V_3.2) is a potential target in trigeminal pain.
TRP channels	DeMartini et al., 2018, Italy [47]	Rat—Chronic constriction injury of the infraorbital nerve	TRPA1 and TRPV1 expression levels were markedly increased post injury.	TRPA1 and TRPV1 channels are potential targets in trigeminal neuropathic pain.
	Santos et al., 2022, Brazil [48]	Mice and rat Formalin injection—Temporomandibular joint. Mustard oil injection—Masseter muscle. Chronic model—Infraorbital nerve transection	Findings showed inhibition of orofacial nociception via TRP channels (TRPV1, TRPM3, TRPM8) in both acute and chronic pain models.	Further studies are needed to check whether the same is effective in females as well. Additionally, TRPV2 and TRPV3 could also be involved.
Serotonin-gated ion channel	Cornelison et al., 2022, MO, USA [49]	Rat—Inflammation induced by injection of Freund’s adjuvant into the trapezius muscle.	Injection of antagonists of the 5-HT3/7 reduced the symptoms.	Explore the role of 5-HT3 in inhibition of trigeminal nociception.
SNP	Katagiri et al., 2012, Japan [50]	Rat—Lingual nerve crush.	Demonstrated evidence that activation of P2Y12R following lingual nerve injury induces neuropathic pain.	Satellite glial cells and trigeminal ganglion neuron interactions are involved in the neuron excitability of lingual nerve crush.
SPR	Raman et al., 2022, Japan [38]	Rat—Infraorbital nerve constriction.	Reported SPR as a novel therapeutic target to treat neuropathic pain	A novel and safe target for new drug designing.
Opioid receptor MOR DOR	Nunez et al., 2007, MD, USA [51]	Rat—Inflammation induced by injection of Freund’s adjuvant into the masseter muscle.	Increased mRNA and protein expression of MOR 3 days post inflammation.	Contributions of MOR in acute and inflammatory muscle pain conditions.
	Erfanparast et al., 2018, Iran [52]	Rat—Inflammation induced by injection of formalin into the vibrissa pad.	Showed the occurrence of biphasic pain post injection.	Involvement of MOR in modulation of antinociception in orofacial region.
	Saloman et al., PA, USA [53]	Rat—Orofacial muscle pain condition induced by capsaicin injection.	Pretreatment with DOR agonist attenuated mechanical hypersensitivity.	Role of DOR in mediation of anti-hyperalgesic response in an acute orofacial muscle pain condition.

VGSC: voltage-gated sodium channel; Na_v_: sodium channel; VGKC: voltage-gated potassium channel; K_v_: potassium channel; VGCC: voltage-gated calcium channel; Ca_v_: calcium channel; TRP: transient receptor potential channels; TRPA: transient receptor potential ankyrin; TRPV: transient receptor potential vanilloid; TRPM: transient receptor potential melastatin; 5-HT: 5-hydroxytryptamine; SNP: single-nucleotide polymorphism; P2Y12R: purinergic receptor P2Y12; SPR: sepiapterin reductase; MOR: Mu opiate receptor; DOR: Delta opiate receptor.

**Table 3:** Potential targets for orofacial pain—clinical trials.

Potential Targets	First Author, Year, Country	Patient Population	Results/Outcome	Opinion
VGSC	Siqueira et al., 2009, Brazil [54]	Trigeminal neuralgia at maxillary and/or mandibular branches. n = 10	Upregulation of Na_v_1.3 and downregulation of Na_v_1.7	The selective expression of particular VGSCs (Na_v_1.3, Na_v_1.7, Na_v_1.8) are suggestive of new targets for drug discovery.
	Zakrzewska et al., 2017, UK [55]	Trigeminal neuralgia patients (based on International Classification of Headache Disorders) (multicenter study) n = 29	The safety and efficacy of Na_v_1.7 in patients with trigeminal neuralgia was assessed. Headache was the most common reported adverse effect.	Needs further clinical trials for effective establishment of Na_v_ 1.7 in patients with trigeminal neuralgia.
	Kotecha et al., 2020, FL, USA [56]	Classical, purely paroxysmal trigeminal neuralgia patients n = 88	A new design for evaluating VGSCs (vixotrigine) for trigeminal neuralgia patients.	Evaluate the pharmacokinetics of sodium channel blocker for an effective treatment.
VGKC	Al-Karagholi et al., 2019, 2020, Denmark [57,58,59]	Migraine patients (1–5 attacks per month) n = 16	The findings from these trials show evidence of migraine attacks in the opening of ATP-sensitive potassium channels.	The role of ATP-sensitive potassium channels and calcium-activated potassium channels (BK_Ca_) in headaches and migraines and the need for a novel drug is evident.
Serotonin-gated ion channel	Christidis et al., 2014, Sweden [60]	Temporomandibular disorder patients n = 5	Increased expression of Na_V_1.8 by 5-HT3A positive nerve fibers.	5-HT3 receptor is a biomarker of myofascial temporomandibular disorders.
SNP	Soeda et al., 2022, Japan [32]	Orofacial pain patients including phantom tooth pain n = 33	The association of SLC17A9 and P2RY12 with the development of phantom tooth pain was evident.	Larger sample size clinical trials would witness the association of these SNPs in phantom tooth pain.
	Slade et al., 2021, NC, USA [61]	Temporomandibular disorder patients with SNP of COMT gene n = 143	COMT alters analgesic efficacy.	It could serve as a precision medicine in treating temporomandibular disorders.
Opioid receptor MOR	Kleinert et al., 2008, Germany [62]	Patients with post-surgical pain after mandibular third molar extraction n = 400	Reported the use of tapentadol, a novel, centrally acting analgesic with two modes of action, combining mu-opioid agonism and norepinephrine. Effectively reduced pain.	Single oral dose with limited side effect.

VGSC: voltage-gated sodium channel; Na_v_: sodium channel; VGKC: voltage-gated potassium channel; ATP: adenosine triphosphate; 5-HT: 5-hydroxytryptamine; SNP: single-nucleotide polymorphism; SLC17A9: Solute carrier family 17 member 9; P2RY12: purinergic receptor P2Y12; COMT: catechol-O-methyltransferase; MOR: Mu opiate receptor.

## Data Availability

Not applicable.
